# Effects of waterlogging at different growth stages on the photosynthetic characteristics and grain yield of sorghum (*Sorghum bicolor* L.)

**DOI:** 10.1038/s41598-023-32478-8

**Published:** 2023-05-03

**Authors:** Ruidong Zhang, Zhongxiao Yue, Xiaofei Chen, Ruidong Huang, Yufei Zhou, Xiong Cao

**Affiliations:** 1 Institute of Industrial Crop, Shanxi Agricultural University, Taiyuan, 030031 Shanxi China; 2 College of Agronomy, Shenyang Agricultural University, Shenyang, 110866 China

**Keywords:** Plant reproduction, Plant stress responses

## Abstract

Various plants, including sorghum (*Sorghum bicolor* L.), are exposed to waterlogging; however, little is known about the effects of waterlogging at different growth stages on sorghum. A pot experiment was conducted using two sorghum hybrids, Jinuoliang 01 (JN01) and Jinza 31 (JZ31), to investigate the effects of waterlogging at different growth stages on the photosynthesis enzyme activity, chlorophyll content, malondialdehyde (MDA) content, photosynthetic parameters, dry matter accumulation, and grain yield. The experiment was conducted using waterlogging treatments implemented at the five-leaf stage (T1), flowering stage (T2), and filling stage (T3), using standard management (no waterlogging) as a control (CK). The adverse effects of waterlogging on sorghum growth varied with the waterlogging timing, with the maximum impact at T1, followed by T2 and T3. JZ31 was more sensitive to waterlogging compared to JN01. Waterlogged conditions inhibited the photosynthetic enzyme activity and reduced the chlorophyll content and photosynthesis, ultimately lowering the biomass yield and grain yield. The maximum yield loss was observed with the T1 waterlogging treatment; the grain yield of JN01 and JZ31 decreased by 52.01–54.58% and 69.52–71.97%, respectively, compared with CK. Furthermore, the decline in grain yield in T1 was associated with reducing grain number per panicle. These findings indicate that sorghum is sensitive to waterlogging at the five-leaf stage and JZ31 is more sensitive to waterlogging than JN01, which may provide a basis for selecting genotypes and management measures to cope with waterlogging in sorghum.

## Introduction

With the increased frequencies of heavy rains under climate change scenario, waterlogging has become one of the most severe abiotic stresses posing selection pressure on agricultural crops^1^. Waterlogging has affected approximately 12% of farmlands^2^, significantly reducing the grain yield^3^. Sorghum (*Sorghum bicolor* L.) is the fifth-largest and widely cultivated food crop in the tropical and subtropical regions. Intermittent or long-term waterlogging due to heavy rains, storms, excessive irrigation, or flooding has affected the sorghum plants of these regions^4^.

Photosynthesis is the most important physiological process of plants that is highly susceptible to waterlogging^5^. Waterlogging also affects the physiological functions of the root system, thereby reducing the plant water status and photosynthesis^6,7^. In plants, stomatal closure is the first response to waterlogging, stomatal closure leads to a restrained decrease in gas exchange, limiting photosynthesis^8,9^. The carbon dioxide (CO_2_) deficiency due to stomatal closure limits the photosynthetic electron transport and increases the reactive oxygen species (ROS) content^10^. The chloroplast is a major source of ROS production but is easily destroyed by ROS^11^. Waterlogging also increases the leaf malondialdehyde (MDA) content and affects the chloroplast membrane lipid peroxidation and integrity^12,13^. Waterlogged conditions at different growth stages affected ribulose-1,5-bisphosphate (RuBP) carboxylase and phosphoenolpyruvate (PEP) carboxylase enzymes^14^, which play significant roles in the C4 pathway of photosynthesis^15^. The activity and content of these enzymes directly affect the photosynthetic rate and the assimilation of CO_2_. Previous studies have also proved that the decline in photosynthesis under waterlogging decreased dry matter accumulation and ultimately grain yield^16–18^. However, little is known about the effects of waterlogging at different growth stages on the photosynthetic enzymes, photosynthetic capacity, and grain yield of sorghum.

The degree of impact of waterlogging varies among genotypes^16,19^ and the timing of waterlogging occurrence^14,20–22^. Luan et al. observed that the waterlogging-tolerant barley had more nodal roots and seminal roots than the sensitive barley^23^. Our previous study found that sorghum genotypes with greater antioxidant capacity and higher net photosynthetic rate exhibited waterlogging tolerance^5^. Waterlogging at different growth stages has different effects on crop growth. Ren et al. confirmed that the adverse effects of waterlogging in the early stages were more serious than those in the later stage^24^. Meanwhile, Zhang et al. observed that waterlogging at squaring had a more pronounced effect on cotton yield than at flowering or boll-setting stage^25^. In wheat, waterlogging that occurs within the stem elongation period had a noticeable impact on yield determination^26^. However, nothing is known on sorghum.

Sorghum faces waterlogging from seedling to maturity. Several studies have reported the effects of waterlogging on the morphology and physiological responses of sorghum^27^; however, few studies have systematically examined the variations in photosynthetic characteristics and grain yield caused by waterlogging at different stages. The study analyzed the effects of waterlogging at different growth stages on the growth, yield, and yield components and the physiological response of two sorghum hybrids.

## Results

### Effects of waterlogging on sorghum yield and yield components

Waterlogging at different growth stages significantly decreased the grain yield of sorghum (Table 1). The grain yield of JN01 decreased by 52.01–54.58%, 18.64–21.24%, and 5.35–8.63%, and that of JZ31 decreased by 69.52–71.97%, 29.30–31.74%, and 18.02–20.91% under T1, T2, and T3, respectively, compared with CK (In 2017 and 2018). In 2017, the grains per panicle under T1 and T2 waterlogging treatments decreased by 57.64% and 20.01%, respectively, in JN01 and by 58.73% and 23.17%, respectively, in JZ31. A similar trend was observed in the grains per panicle in 2018. Besides, the adverse effects of waterlogging on 1000-grain weight (TGW) varied with the treatments and hybrids. The T1 treatment resulted in the most significant TGW reduction for JZ31 (24.69% and 25.75% in 2017 and 2018, respectively).

**Table 1:** Effects of waterlogging at different growth stages on the yield and yield components of sorghum.

Year	Hybrid	Treatment	Panicles ha^–1^	Grains panicle^–1^	1,000-grain weight (g)	Grain yield (kg ha^–1^)
2017	JN01	CK	64,092.1 ± 1084.7 a	4904.2 ± 248.7 a	20.06 ± 0.68 b	6300.94 ± 365.31 a
		T1	62,983.0 ± 1701.8 a	2077.0 ± 146.1 c	21.92 ± 0.93 a	2861.80 ± 222.72 c
		T2	62,958.9 ± 2809.4 a	3922.8 ± 173.7 b	20.06 ± 0.52 b	4962.73 ± 671.96 b
		T3	62,840.6 ± 2180.3 a	4757.3 ± 207.5 a	19.25 ± 0.41 b	5757.27 ± 607.56 a
	JZ31	CK	62,776.5 ± 2821.5 a	2845.7 ± 146.1 a	32.24 ± 1.32 a	5766.95 ± 614.84 a
		T1	61,793.9 ± 1429.6 a	1174.2 ± 100.9 c	24.28 ± 1.02 c	1757.91 ± 178.95 d
		T2	62,561.3 ± 2093.9 a	2186.3 ± 103.9 b	29.80 ± 0.90 b	4077.19 ± 418.24 c
		T3	63,638.1 ± 2030.5 a	2911.7 ± 103.7 a	25.52 ± 0.66 c	4727.67 ± 342.69 b
2018	JN01	CK	65,011.1 ± 1223.9 a	4915.7 ± 242.3 a	20.03 ± 0.60 b	6394.01 ± 219.26 a
		T1	63,873.2 ± 1858.5 a	2013.1 ± 141.1 c	23.88 ± 0.57 a	3068.65 ± 216.49 c
		T2	63,907.7 ± 1725.1 a	3986.8 ± 175.1 b	20.42 ± 0.30 b	5202.46 ± 260.65 b
		T3	63,770.4 ± 1692.7 a	4835.1 ± 199.9 a	19.62 ± 0.45 b	6052.89 ± 384.38 a
	JZ31	CK	63,736.3 ± 1957.0 a	3006.9 ± 155.0 a	33.32 ± 0.70 a	6382.90 ± 327.70 a
		T1	62,896.4 ± 1234.1 a	1151.8 ± 71.8 c	24.74 ± 0.97 c	1788.89 ± 63.51 d
		T2	63,106.0 ± 1959.1 a	2248.2 ± 126.8 b	30.69 ± 0.62 b	4357.05 ± 343.26 c
		T3	64,505.5 ± 1977.8 a	3018.7 ± 53.0 a	25.91 ± 0.51 c	5048.25 ± 270.05 b
ANOVA						
Year (Y)			NS	NS	**	*
Hybrid (H)			NS	**	**	**
Treatment (T)			NS	**	**	**
Y × H			NS	NS	NS	NS
Y × T			NS	NS	NS	NS
H × T			NS	**	**	**
Y × H × T			NS	NS	*	NS

Different lowercase letters within a column indicate significant differences (*P* < 0.05, Duncan’s multiple range test). NS, not significant; *, significant at the 0.05 probability level; **, significant at the 0.01 probability level.

### Effects of waterlogging on sorghum biological yield and harvest index

Waterlogging had negative effects on sorghum biomass yield and harvest index, which varied with the genotypes and the growth stages (Table 2). Waterlogging significantly reduced the biomass yield. In JN01, T1 waterlogging treatment reduced the biomass yield by 55.29%, followed by T2 and T3 (18.13% and 9.05%, respectively). The biomass yield decrease in JZ31 was 63.99%, 32.92%, and 13.91%, under T1, T2, and T3, respectively, compared with the CK. Meanwhile, the harvest index decreased by 21.06% and 9.48% under T1 and T3 in JZ31; however, no significant difference was observed in JN01 (Table 2).

**Table 2:** Effects of waterlogging at different stages on the plant biomass and harvest index of sorghum (2018).

Hybrids	Treatments	Plant biomass (g)	Harvest index (%)
JN01	CK	247.58 ± 12.40 a	39.39 ± 2.38 a
	T1	120.69 ± 5.43 d	39.82 ± 1.47 a
	T2	202.69 ± 7.84 c	40.18 ± 1.43 a
	T3	225.18 ± 8.54 b	42.19 ± 2.76 a
JZ31	CK	256.22 ± 18.82 a	39.19 ± 2.05 a
	T1	92.26 ± 5.66 d	30.93 ± 2.29 c
	T2	171.87 ± 9.48 c	40.21 ± 2.90 a
	T3	220.59 ± 4.52 b	35.47 ± 1.20 b
ANOVA			
Hybrid (H)		**	NS
Treatment (T)		**	**
H × T		**	NS

Different lowercase letters within a column indicate significant differences (*P* < 0.05, Duncan’s multiple range test). NS, not significant; *, significant at the 0.05 probability level; **, significant at the 0.01 probability level.

### Effects of waterlogging on sorghum panicle traits

The panicle length, panicle width, and grain weight per panicle of both the hybrids decreased significantly under waterlogging. The maximum effects were observed in T1, followed by T2 and T3. The panicle length, panicle width, and grain weight per panicle of JN01 in T1 decreased by 14.70%, 30.77%, and 51.11%, respectively, while those of JZ31 decreased by 33.85%, 51.02%, and 71.62%, respectively, compared with the corresponding CKs. Besides, waterlogged conditions affected the panicle differentiation in both hybrids, especially under T1. Compared to CK, the waterlogging treatment T1 reduced the number of primary branches and secondary branches by 17.80% and 9.52%, respectively; the number of seeds in the primary and secondary branches by 50.62% and 45.45%, respectively, in JN01. In JZ31, a higher decrease was observed (21.25%, 35.71%, 51.19%, and 28.13%, respectively) (Table 3).

**Table 3:** Effects of waterlogging at different growth stages on the morphological characteristics of sorghum panicle (2018).

Hybrids	Treatments	Panicles length (cm)	Panicle width (cm)	Grains weight per panicle (g)	No. of primary branch	No. of seeds in the primary branch	No. of secondary branch	No. of seeds in the secondary branch
JN01	CK	34.7 ± 0.8 a	9.1 ± 0.7 a	98.34 ± 2.02 a	76.4 ± 3.5 a	64.8 ± 4.0 a	4.2 ± 0.4 b	15.4 ± 0.9 a
	T1	29.6 ± 2.0 c	6.3 ± 0.9 b	48.08 ± 3.63 c	62.8 ± 4.7 b	32.0 ± 2.0 c	3.8 ± 0.4 b	8.4 ± 0.5 c
	T2	32.7 ± 0.7 b	8.1 ± 1.8 a	81.39 ± 2.91 b	73.8 ± 2.8 a	53.8 ± 1.3 b	3.8 ± 0.4 b	14.0 ± 1.2 b
	T3	34.6 ± 0.6 a	8.7 ± 0.3 a	94.86 ± 4.10 a	74.8 ± 2.3 a	64.4 ± 1.1 a	4.2 ± 0.4 b	15.2 ± 0.8 a
JZ31	CK	32.2 ± 0.8 a	9.8 ± 0.5 a	100.22 ± 6.04 a	89.4 ± 2.7 a	33.6 ± 1.9 a	5.6 ± 0.5 a	6.4 ± 0.5 a
	T1	21.3 ± 2.0 d	4.8 ± 0.2 c	28.44 ± 0.91 d	70.4 ± 4.8 b	16.4 ± 1.1 c	3.6 ± 0.5 b	4.6 ± 0.5 b
	T2	26.7 ± 1.0 c	8.0 ± 0.5 b	69.01 ± 4.43 c	87.0 ± 7.6 a	26.0 ± 1.6 b	5.0 ± 0.6 a	5.4 ± 0.5 ab
	T3	29.3 ± 1.4 b	8.5 ± 0.9 b	78.22 ± 2.10 b	87.2 ± 3.7 a	34.6 ± 1.3 a	5.6 ± 0.5 a	6.2 ± 0.4 a
ANOVA								
(H)		**	NS	**	**	**	**	**
(T)		**	**	**	**	**	**	**
H × T		**	NS	**	**	**	**	**

Different lowercase letters within a column indicate significant differences (*P* < 0.05, Duncan’s multiple range test). NS, not significant; *, significant at the 0.05 probability level; **, significant at the 0.01 probability level.

### Effects of waterlogging on chlorophyll content and MDA content in sorghum leaves

**Figure 1:**
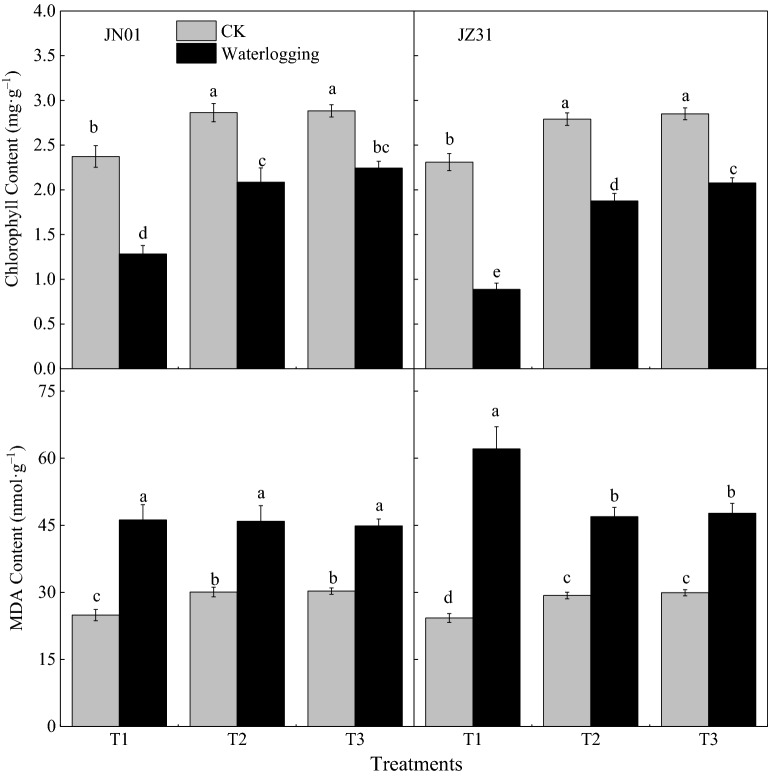
Effects of waterlogging at different stages on the chlorophyll content and MDA content of sorghum leaves (2018). *Note* T1 refers to waterlogging at the five-leaf stage; T2 refers to waterlogging at the onset of flowering; T3 refers to waterlogging at the grain-filling stage; and the indicators of CK were measured, along with the different treatments. Means and standard errors based on three replicates are shown. Means followed by the same letter within a column do not significantly differ at *P* < 0.05 according to Duncan’s Multiple Range Test.

Waterlogging affected the chlorophyll content of sorghum leaves (Fig. 1). The T1, T2, and T3 treatments decreased the chlorophyll content in sorghum leaves of JN01 by 45.93%, 27.12%, and 22.20%, respectively, and in JZ31 by 61.62%, 32.74%, and 27.13%, respectively, compared with the corresponding controls. However, MDA content in sorghum leaves increased after waterlogging (Fig. 1). Under T1, T2, and T3, the MDA content of JN01 was 1.85-fold, 1.53-fold, and 1.48-fold higher than that of CK, and in JZ31 was 2.56-fold, 1.60-fold, and 1.59-fold higher than that of the corresponding CKs.

### Effects of waterlogging on RuBP carboxylase and PEP carboxylase activity in sorghum leaves

**Figure 2:**
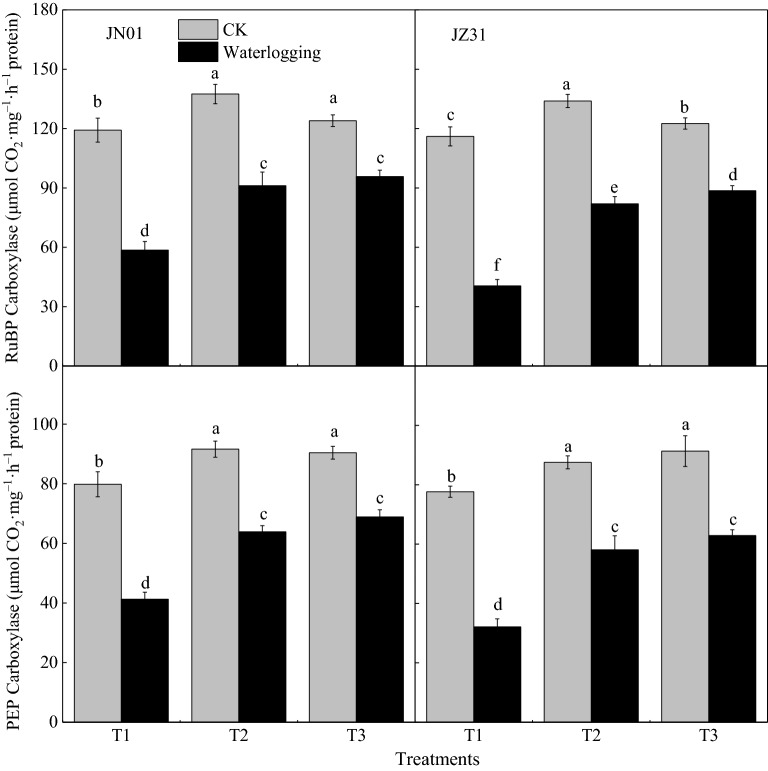
Effects of waterlogging at different stages on the activity of RuBP carboxylase and PEP carboxylase in sorghum leaves (2018). *Note* T1 refers to waterlogging at the five-leaf stage; T2 refers to waterlogging at the onset of flowering; T3 refers to waterlogging at the grain-filling stage; and the indicators of CK were measured, along with the different treatments. Means and standard errors based on three replicates are shown. Means followed by the same letter within a column do not significantly differ at* P* < 0.05 according to Duncan’s Multiple Range Test.

RuBP carboxylase and PEP carboxylase activity of the two hybrids decreased under waterlogging at different growth stages (Fig. 2). The most significant reduction in the RuBP carboxylase activity was observed in T1, followed by T2 and T3. The JZ31 showed higher negative effects. RuBP carboxylase activity of JN01 decreased by 50.82%, 33.68%, and 22.77%, and that of JZ31 by 65.09%, 38.79%, and 27.68% in T1, T2, and T3, respectively, compared with the corresponding CKs. The effect of waterlogging on PEP carboxylase activity was similar to that of RuBP carboxylase activity. The treatments T1, T2, and T3 decreased the PEP carboxylase activity by 48.30%, 30.25%, and 23.78%, respectively, in JN01 and 58.64%, 33.63%, and 31.12%, respectively, in JZ31, compared with the corresponding CKs.

### Effects of waterlogging on photosynthetic parameters

**Figure 3:**
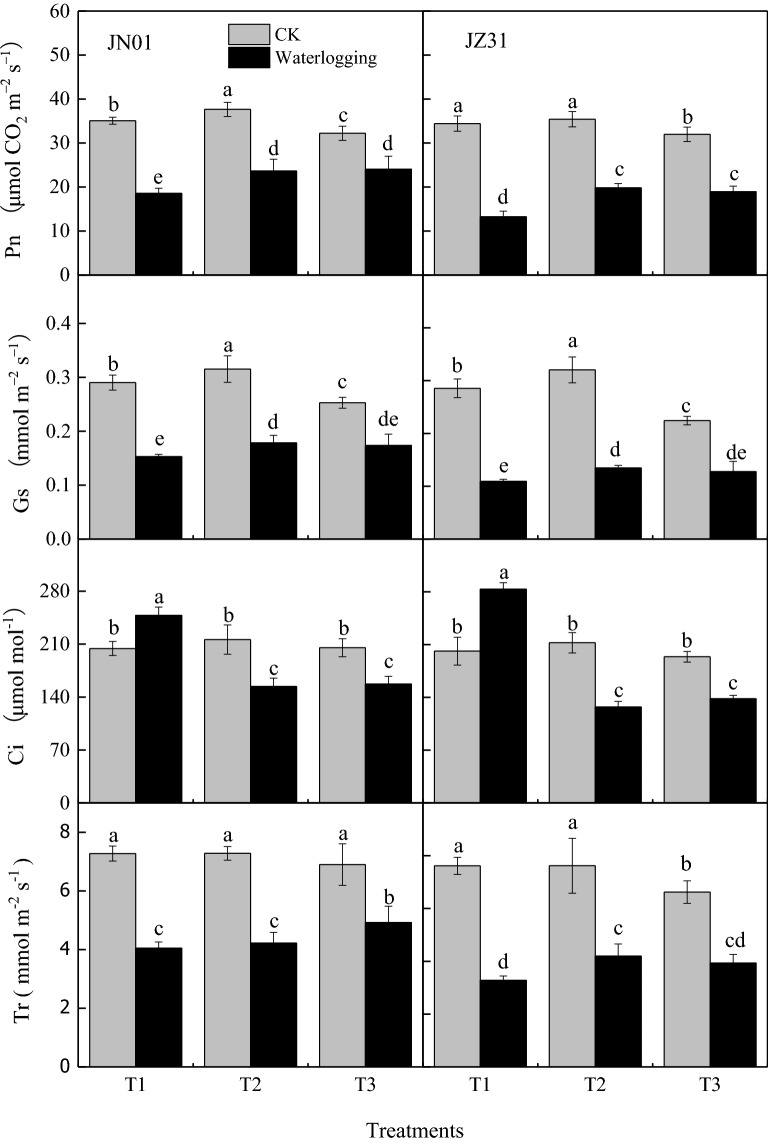
Effects of waterlogging at different stages on the photosynthetic parameters of sorghum leaves (2018). *Note* T1 refers to waterlogging at the five-leaf stage; T2 refers to waterlogging at the onset of flowering; T3 refers to waterlogging at the grain-filling stage; and the indicators of CK were measured, along with the different treatments. Means and standard errors based on five replicates are shown. Means followed by the same letter within a column do not significantly differ at *P* < 0.05 according to Duncan’s Multiple Range Test.

Further, the effects of waterlogging on sorghum photosynthesis in both hybrids were analyzed. The photosynthetic parameters, such as the net photosynthetic rate (Pn), stomatal conductance (Gs), and transpiration rate (Tr), significantly decreased under waterlogging (Fig. 3). The most significant effects were observed in T1, followed by T2 and T3 in both the hybrids. After the waterlogging treatment T1, the Pn, Gs, and Tr of JN01 decreased by 47.04%, 47.34%, and 44.32%, respectively, and JZ31 by 61.54%, 61.73%, and 56.98%, respectively, compared with the corresponding CKs. Meanwhile, T1 increased the intercellular CO_2_ concentration (Ci) (JN01, 1.21-fold and JZ31, 1.41-fold), whereas T2 and T3 reduced (JN01, 28.74% and 23.43% and JZ31, 40.11% and 28.71%), compared with the corresponding CKs.

## Discussion

This study examined the effects of waterlogging at the five-leaf, flowering, and filling stages on photosynthesis and grain yield in sorghum. The different waterlogging treatments reduced the grain yield to different levels; the maximum decrease was found when waterlogging occurred at the five-leaf stage (T1). Studies have reported that waterlogging at the three-leaf (V3) stage in summer maize resulted in maximum grain yield loss^30^, followed by the six-leaf (V6) and tasseling (VT) stages^14^. Consistent with these observations in maize, the present study also demonstrated that waterlogging at the early growth stage caused severe damage to sorghum grain yield than that at the reproductive stage.

Under waterlogging, the yield components of sorghum, especially grain number per panicle and TGW, got easily affected. Data from the present experiment confirmed that waterlogging at the five-leaf stage and flowering stage (T1 and T2) resulted in a significant decrease in grain number per panicle in both the hybrids, similar to the decline in spikes per plant observed in barley under waterlogging^31^. This decrease in grain number was consistent with the decrease in panicle length and width. Grain number is a genotype- and environment-dependent trait influenced by crop cycle, from germination to maturity^16^. In this study, the maximum negative effect of waterlogging on grain number was found in T1, probably because waterlogging at the five-leaf stage affected subsequent panicle differentiation. Reddy and Mittrain observed a similar phenomenon in rice^22^ and De San Celedonio et al. in barley^16^. The reduction in grain number was associated with panicle size and panicle weight. However, the change in panicle structure due to waterlogging was different between the two hybrids. Under waterlogged conditions, the reduction in grain number in JN01 was mainly due to the decrease in the number of seeds in the secondary branches, while that in JZ31 was mainly due to the reduction in the number of secondary branches. Besides, the negative effects of waterlogging on the TGW of JN31 was more evident than that of JN01. Thus, the differences in panicle structure may reflect the differences in tolerance to waterlogging in sorghum.

The accumulation and distribution of photosynthetic products are the major factors that determine grain yield. Waterlogging adversely affects biomass accumulation and partitioning to reproductive organs, resulting in decreased grain yield and harvest index^20,32^. The loss in cotton yield under waterlogging was mainly attributed to the more significant reduction in biological yield, harvest index, and boll density and weight^25^. In this experiment, waterlogged conditions decreased grain yield in both hybrids by reducing biomass yield and harvest index (although in lesser magnitude for the latter), consistent with the previous reports in maize^20^ and barley^31^. Waterlogging at the five-leaf stage led to the maximum reduction in the biomass, indicating it as the critical period for sorghum. We speculate that the sorghum plants thrive during this period, and waterlogging in T1 slowed down sorghum growth and development, significantly reducing biomass yield.

The decrease in biomass mainly occurs due to limited photosynthesis. The photosynthesis of sorghum leaves is easily affected by waterlogging stress. In this experiment, waterlogged conditions restricted the gas exchange through the leaf stomata, in agreement with the reduced photosynthetic parameters, including Pn, Gs, and Tr, in maize^33^ and pepper^34^. Thus, the present study indicated that waterlogging restricted leaf photosynthesis and the photosynthetic assimilation capacity of sorghum. The maximum reduction was found in T1, which means a pronounced effect of waterlogging was observed on biomass yield when it occurred at the early stage. The chlorophyll content is an important indicator reflecting the crop photosynthetic capacity^35^. Previous studies have demonstrated significantly low leaf chlorophyll content under waterlogged conditions, especially in sensitive plant species such as cotton^25,36^, maize^21^ and wheat^37^. In sorghum also, waterlogging decreased the chlorophyll content and reduced the photosynthesis, accelerating the senescence process. Besides, photosynthetic enzymes such as RuBP carboxylase and PEP carboxylase affect the photosynthetic rate and CO_2_ assimilation^38^. In this study, waterlogging had a pronounced effect on the activity of these photosynthetic enzymes. The RuBP carboxylase and PEP carboxylase activities declined under waterlogging in T1, T2, and T3. Therefore, waterlogging reduced the photosynthetic enzyme activity in sorghum, decreasing photosynthesis, consistent with the previous studies in maize^14^. The reduction in chlorophyll content and photosynthetic enzyme activity was also correlated with ROS, which caused oxidative membrane damage, resulting in MDA accumulation^10^. In the present experiment, an obvious increase in the MDA content of sorghum leaves was observed in response to waterlogging stress, and the highest increase was found in T1. Similar results were reported by Yordanova et al.^39^ and Yu et al.^40^. The changes in chloroplast morphology under waterlogging were associated with increased active oxygen content^23^ and damaged protective enzyme system^12,41^. This study indicated that waterlogging destroyed the integrity of cells, reduced the activity of photosynthetic enzymes, reduced the content of chlorophyll, and ultimately leads to a decline in photosynthesis. Waterlogging at the five-leaf stage (T1) caused severe damage.

## Conclusion

The study demonstrated that waterlogging caused a significant decline in sorghum grain yield. Sorghum was the most sensitive to waterlogging at the five-leaf stage, followed by flowering and filling stages. Between the hybrids of the study, JZ31 was more sensitive to waterlogging than JN01. The sorghum grain yield loss occurred mainly because waterlogging repressed the photosynthetic enzyme activity and decreased the chlorophyll content and photosynthesis, ultimately reducing the biomass yield and grain number per panicle. This study explains the differences in waterlogging effects with stages and genotypes in sorghum, which will help propose measures to alleviate or avoid the stress.

## Materials and methods

### Plant materials and cultivation

Two sorghum hybrids, Jinuoliang 01 (JN01), tolerant to waterlogging, and Jinza 31 (JZ31), sensitive to waterlogging^8^, were used in this experiment. A pot culture experiment was conducted at the experimental farm of Shenyang Agriculture University, China (41°49′ N and 123°33′ E) in 2017 and 2018. The experimental farm is located in the northern temperate zone and has a subhumid continental climate. The annual frost-free period was 155–180 days, and the mean annual temperature was 8 °C. The average precipitation was 716.2 mm, and the rainfall mainly occurred from June to August, often in the form of torrential rain^28^. Pots (33 cm diameter and 30 cm height) with three holes at the bottom to drain off excess water were filled with 19 kg loam soil obtained from the nearby farmland. The soil had 18.02 g kg^−1^ of organic matter, 0.92 g kg^−1^ of total nitrogen, 58.67 mg kg^−1^ of rapidly available phosphorus, and 80.26 mg kg^−1^ of rapidly available potassium. Sorghum seeds were sown on May 14 in 2017 and May 11 in 2018.

### Experimental design and treatment

The experiment was performed using four treatments from 2017 to 2018: T1, waterlogging at the five-leaf stage; T2, waterlogging at the onset of flowering; T3, waterlogging at the grain-filling stage, each treatment was waterlogged for 14 days. CK, without waterlogging, optimal moisture conditions (75–80% soil moisture) were maintained over the entire growth stage, Soil water content was measured by using soil moisture measuring instrument (TDR100, Spectrum, USA). The experiment was carried out in a completely randomized design using five replicates per treatment. The plants were grown in pots to ensure that the plants at different stages were stressed in the same manner under the same growing conditions. Each pot was placed into another pot lacking drainage holes for the waterlogging treatments, and the water level was retained at 3 cm above the soil surface within the inner pot. Following local management measures, disease, weeds, and pests were well controlled.

### Sampling and measurements

#### Photosynthetic parameters

One day after the end of each waterlogging treatment, the latest fully expanded leaf in T1 and the flag leaves in T2 and T3 were selected to measure the photosynthetic parameters. The photosynthetic parameters, including Pn, Tr, Gs, and Ci, were measured using a Li-6400 portable photosynthesis system (LI − COR, Lincoln, NE, USA), following a previously Ren et al. method^13^. The photosynthetically active radiation (PAR) from the LED light source was set as 1600 μmol m^−2^, and CO_2_ concentration at 360 μmol mol^−1^ through a CO_2_ buffer bottle during the measurement. Five plants per treatment were randomly selected to measure the photosynthesis parameters, generally done between 10:00 AM to 12:00 PM.

The leaves used for assaying chlorophyll content, MDA content, and photosynthetic enzymes activity were the same as those used to measure the photosynthetic parameters. After measuring the photosynthetic parameters, the leaves were harvested, frozen in liquid nitrogen, and stored at − 80 °C for further analysis.

#### Chlorophyll content

Approximately 0.2 g of the sampled leaf was cut into small pieces with scissors, excluding the main veins. The leaf pieces were immersed in alcohol (96%, v/v) and kept at 4 °C in the dark until it turned white. The absorbance of the alcohol extract was determined at 649 and 665 nm using a UV-spectrophotometer (Hitachi UV-1800, Kyoto, Japan). Chlorophyll extraction and calculation of chlorophyll content were according to the method by Zhang et al.^5^.

#### MDA content

Approximately 0.5 g of the leaf tissue was homogenized in 5 mL of trichloroacetic acid (5%, v/v) and centrifuged at 5000× *g* for 20 min at 4 °C. The supernatant (1.5 mL) was mixed with 2.5 mL of 5% trichloroacetic acid containing 0.5% 2-thiobarbituric acid, and the mixture was heated at 100 °C for 15 min. The reaction mixture was cooled to room temperature and centrifuged at 5000× *g* for 10 min. The absorbance of the supernatant was determined at 532 and 600 nm with a UV-spectrophotometer. The MDA content was calculated following the Dionisio-Sese and Tobita method^29^.

#### Photosynthetic enzyme activity

Approximately 0.5 g of the leaf tissue was ground with a buffer containing 0.1 mol·L^−1^ Tris–HCl (pH 7.8), 1 mmol·L^−1^ EDTA, 7 mmol·L^−1^ mercaptoethanol, and 10% glycerol to obtain the extract. This extract was centrifuged at 15,000× *g* for 30 min at 4 °C. The supernatant was used to determine the enzyme activity. The activity of ribulose-1,5-bisphosphate carboxylase (RuBP Case) and phosphoenolpyruvate carboxylase (PEP Case) was measured using assay kits (Nanjing Jiancheng Bioengineering Institute, China), following the manufacturer’s instructions.

#### Grain yield and biomass yield

At maturity, five shoots were harvested from each treatment to determine the grain yield and biomass yield. The panicle was separated from the plant, and the length and width of the panicles were measured using a ruler. The number of primary and secondary branches in each sorghum panicle and seeds on the primary and secondary branches was determined. The shoots, including the stem, the leaf, and the panicle, were dried at 80 °C to constant weight and weighed to determine the biomass yield. Then, 1000 grains were counted from each panicle and weighed to determine the TGW.

$$ \begin{aligned} {\text{Grain }}\,{\text{yield}}\, \, \left( {{\text{kg ha}}^{{ - {1}}} } \right) & = {\text{Harvested}}\,{\text{ panicles}}\, \, \left( {{\text{panicles}}\,{\text{ ha}}^{{ - {1}}} } \right) \, \\ & \;\;\;\; \times {\text{ Grain}}\,{\text{ number}}\,{\text{ panicle}}^{{{-}{1}}} \times { 1}000 - {\text{grain}}\,{\text{ weight}}\, \, \left( {{\text{g 1}}000\,{\text{ grains}}^{{{-}{1}}} } \right) \, \times { 1}0^{{ - {6}}} \\ {\text{Harvest}}\,{\text{ index}} & = {\text{ Grain}}\,{\text{ yield}}/{\text{Total}}\,{\text{ biomass }}\,{\text{yield}} \times {1}00\% . \\ \end{aligned} $$

### Statistical analysis

Data were subjected to analysis of variance (ANOVA) in SPSS (Ver.17.0, SPSS, Chicago, IL, USA). Duncan’s multiple range test was performed to estimate the significant differences among the treatments at the 0.05 probability level (*P* < 0.05). The figures were generated using Origin 8.0 (Origin Lab Corporation, USA).

## Supplementary Information

Supplementary Information.

## Data Availability

All methods were performed following the relevant guidelines and regulations. All data generated or analyzed during this study are included in this published article and its supplementary information files.
